# Pharmacokinetics of the most commonly used antihypertensive drugs throughout pregnancy methyldopa, labetalol, and nifedipine: a systematic review

**DOI:** 10.1007/s00228-022-03382-3

**Published:** 2022-09-15

**Authors:** Dylan van de Vusse, Paola Mian, Sam Schoenmakers, Robert B. Flint, Willy Visser, Karel Allegaert, Jorie Versmissen

**Affiliations:** 1 Department of Internal Medicine, Division of Pharmacology and Vascular Medicine, Erasmus MC University Medical Center, PO Box 2040, 3000 CA Rotterdam, The Netherlands; 2 Department of Clinical Pharmacy, Erasmus MC University Medical Center, PO Box 2040, 3000 CA Rotterdam, The Netherlands; 3 Clinical Pharmacy and Pharmacology, University Medical Center Groningen, Groningen, The Netherlands; 4 Obstetrics and Gynecology, Division Obstetrics and Prenatal Medicine, Erasmus MC University Medical Center, Rotterdam, The Netherlands; 5 Pediatrics, Division of Neonatology, Erasmus MC University Medical Center, Rotterdam, The Netherlands; 6 Department of Development and Regeneration, KU Leuven, Leuven, Belgium; 7 Department of Pharmaceutical and Pharmacological Sciences, KU Leuven, Leuven, Belgium

**Keywords:** Methyldopa, Labetalol, Nifedipine, Pharmacokinetics, Pregnancy, Hypertension, Hypertensive disorders of pregnancy

## Abstract

**Purpose:**

Antihypertensive drugs are among the most prescribed drugs during pregnancy. Methyldopa, labetalol, and nifedipine have been perceived safe to use during pregnancy and are therefore recommended in international guidelines for treatment of hypertension. In this review, we provide a complete overview of what is known on the pharmacokinetics (PK) of the antihypertensive drugs methyldopa, labetalol, and nifedipine throughout pregnancy.

**Methods:**

A systematic search was performed to retrieve studies on the PK of methyldopa, labetalol, and nifedipine used throughout pregnancy. The search was restricted to English and original studies. The systematic search was conducted on July 27, 2021, in Embase, Medline Ovid, Web of Science, Cochrane Library, and Google Scholar. Keywords were methyldopa, labetalol, nifedipine, pharmacokinetics, pregnancy, and placenta.

**Results:**

A total of 1459 unique references were identified of which title and abstract were screened. Based on this screening, 67 full-text papers were assessed, to retain 30 PK studies of which 2 described methyldopa, 12 labetalol, and 16 nifedipine. No fetal accumulation is found for any of the antihypertensive drugs studied.

**Conclusion:**

We conclude that despite decades of prescribing methyldopa, labetalol, and nifedipine throughout pregnancy, descriptions of their PK during pregnancy are hampered by a large heterogeneity in the low number of available studies. Aiming for evidence-based and personalized dosing of antihypertensive medication in the future, further studies on the relationship of both PK and pharmacodynamics (including the optimal blood pressure targeting) during pregnancy and pregnancy-related pathology are urgently needed to prevent undertreatment, overtreatment, and side effects.

**Supplementary Information:**

The online version contains supplementary material available at 10.1007/s00228-022-03382-3.

## Introduction

Pregnant women frequently use prescription and over-the-counter drugs: more than 80% use at least one drug throughout pregnancy including folic acid to reduce neonatal mortality and morbidity from neural tube disorders; 1.5–2% use cardiovascular drugs [1–5]. Information on optimal dosing of drugs throughout pregnancy is widely lacking, as ethical, legal, and practical reasons often prevent inclusion of pregnant women in clinical trials [6]. As a result, most drug use during pregnancy is “off-label” even when commonly used [7, 8]. Recently, the same issue occurred during the COVID-19 pandemic in which initially information on COVID-19 vaccination throughout pregnancy was lacking [9, 10]. An international call has been made to start including pregnant women in clinical trials instead of excluding them [11].

Pregnancy is associated with physiological changes, like body composition, renal clearance, protein concentration, and enzyme activity that has been shown to significantly alter the pharmacokinetics (PK) of many drugs, like amoxicillin and antipsychotics [12–14]. Due to these PK changes, simple extrapolation of normal dosages for nonpregnant women to dosages for pregnant women may lead to either subtherapeutic drug effects or supratherapeutic exposure in expecting women or toxic effects in the fetus due to placental transfer [15]. Furthermore, drug use can lead to placental dysfunction or accumulation of the drug, fetal (over)exposure, and teratogenic side effects. As an appropriate dose of a drug during pregnancy for a specific indication can be difficult to determine, most dosages have been derived empirically. In general, the aim is to dose as low as possible [16].

Hypertension is one of the most common health problems among pregnant women for which pharmacotherapy is indicated [17]. The incidence of hypertension for primigravida and multigravida is 10–15% and 2–5%, respectively [18]. Hypertensive disorders during pregnancy are classified as follows according to the International Society for the Study of Hypertension in Pregnancy (ISSHP): chronic hypertension (predating or diagnosed before 20 weeks of pregnancy), gestational hypertension (de novo after 20 weeks of gestation), (pre)eclampsia (de novo or superimposed on chronic hypertension: hypertension after 20 weeks of gestation accompanied by proteinuria and/or evidence of maternal acute kidney injury, liver dysfunction, neurological features, hemolysis or thrombocytopenia, or fetal growth restriction). Adequate and early treatment of hypertension during pregnancy is of major importance, as severe hypertension is associated with an increased risk of (pre)eclampsia and hemolysis, elevated liver enzymes, and a low platelet count (HELLP) syndrome, leading to an increased maternal and perinatal morbidity and mortality [19]. It should be noted that it has not been proven that blood pressure control lowers this risk [20]. Commonly used antihypertensive drugs in younger patients angiotensin-converting enzyme (ACE) inhibitors or angiotensin receptor blockers (ARBs) are contraindicated during pregnancy because of increased risk for fetal renal damage, which seems even higher after exposure to ARBs than that to ACE inhibitors [21]. Currently, methyldopa, labetalol, and nifedipine are considered safe to use during pregnancy and therefore recommended in guidelines [22] [23]. However, for example, beta-blocker use has been associated with hypoglycaemia, which, although rare, can cause severe brain injury in neonates postpartum upon exposure via placental transfer [24, 25]. Furthermore, beta-blocker exposure during pregnancy has been associated with preterm birth, newborns small for gestational age, and perinatal mortality [26, 27]. However, labetalol, a beta-blocker with also alpha blockade, has not been associated with the adverse neonatal outcomes that other beta-blockers are associated with. The mechanisms of action and metabolic routes of methyldopa, labetalol, and nifedipine in nonpregnant adults are described in Supplementary Table 1 [28–39]. Nevertheless, evidence-based PK adjusted dosages for optimizing safe and effective treatment of hypertension in pregnant women are yet lacking. Methyldopa, labetalol, and nifedipine are considered safe to use during pregnancy, and therefore, the fetal/maternal ratio of these drugs is expected < 1, which means that fetal accumulation does not occur.

The aim of this systematic review was to generate a complete overview of the evidence on the PK of the antihypertensive drugs methyldopa, labetalol, and nifedipine throughout pregnancy, after which knowledge gaps were identified. Our ultimate goal was to direct future research aiming for personalized medicine dosing of these drugs in pregnant women.

## Methods

### Protocol and registration

The protocol for this systematic review was registered in the International prospective register of systematic reviews (PROSPERO) and published on April 11, 2019 (registration number: CRD42019128415) [40]. To improve the reporting of this systematic review, the PRISMA checklist was used [41].

### Eligibility criteria

A systematic search was performed to retrieve studies on the PK throughout pregnancy of methyldopa, labetalol, and nifedipine. Participants included pregnant women of all gestational ages (when possible) compared with nonpregnant women or women in the postpartum period. The outcome of a study should include plasma concentration–time data and/or PK parameters (e.g., elimination half-life (t_1/2_), clearance (CL), volume of distribution (V_d_), area under the plasma drug concentration–time curve (AUC), maximum serum concentration (C_max_), and time at which the maximum serum concentration was reached (t_max_)) in pregnant women. Ex vivo human placental perfusion model studies were also eligible for inclusion. Furthermore, amniotic fluid and fetal (umbilical cord) blood drug concentrations were included in this systematic review due to its relevance for describing fetal PK. Drug fractions in the amniotic fluid may have been renally cleared by the fetus to the amniotic cavity, and can be reabsorbed by swallowing the amniotic fluid. Eligible studies were randomized controlled trials, observational studies, and case reports. No publication date restrictions were applied. The search was restricted to English studies only.

### Search strategy

The systematic search was conducted on August 2, 2022, in Embase, Medline Ovid, Web of Science, Cochrane Library, and Google Scholar. Keywords were methyldopa, labetalol, nifedipine, pharmacokinetics, pregnancy, and placenta. The detailed search strategy is outlined in Appendix 1. References of included studies were checked for relevant studies to be potentially included in this systematic review.

### Study selection and data extraction

Titles and abstracts of the retrieved studies were screened for relevance, after which full-texts of potentially eligible studies were obtained. Studies not meeting the study aim and inclusion criteria were excluded. The study selection was performed independently by two investigators (DV, PM). In case of disagreement, a third author (JV) was consulted. Data extraction from the reports was performed independently by two investigators (DV, PM) and verified on similarity.

## Results

### Study selection and data extraction

**Fig. 1:**
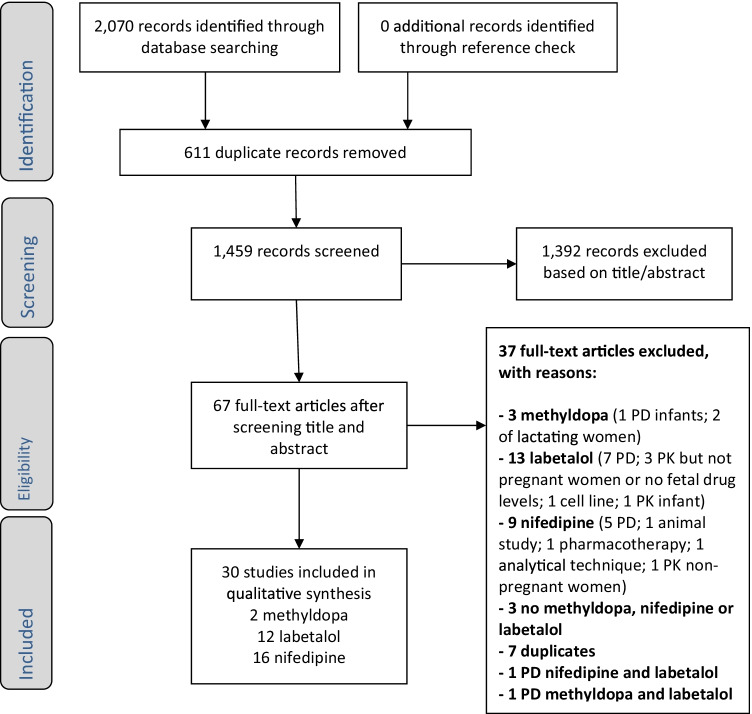
Flow diagram screened studies. Abbreviations: PD, pharmacodynamics; PK, pharmacokinetics

A total of 2070 records were identified. After removal of duplicates, a total of 1459 references remained of which title and abstract were screened (Fig. 1). Based on this screening, full-texts were assessed for 67 studies, to retain 30 included PK studies: 2 on methyldopa, 12 on labetalol, and 16 on nifedipine. The most common reason for exclusion was that studies investigated pharmacodynamics (PD) only and PK data were not available. No additional records were found from references of included studies.

### Study characteristics

Table 1 provides the characteristics of the 30 included PK studies. The number of subjects per study ranged from 9 to 12 for methyldopa, 1 to 57 for labetalol, and 5 to 40 for nifedipine. Three out of the 30 included studies were physiologically based pharmacokinetics (PBPK) studies, all three concerning nifedipine. One study reported data of the first trimester, three of the second trimester, twenty-two of the third trimester, and nine around delivery. The PK parameters of methyldopa, labetalol, and nifedipine derived from literature are provided in Tables 2, 3, 4, and 5, respectively. Fetal side effects were not present [42] or (in most studies) not described.

**Table 1:** Study characteristics of the included studies. Studies are ordered by drug, indication/type of study, and year

**Ref**	**Study indicated by author (year of publication)**	**Drug**	**Country**	**Dose**	**Route of administration**	**Indication (or ex vivo)**	**Number of subjects**	**Samples**	**Gestational age (weeks)**	**Statistical approach**
[43]	**Jones HM et al. (1978)**	Methyldopa	UK	750–2000 mg/day	Oral	Hypertension	12	30	Unknown	Other***
[44]	**Jones HM et al. (1979)**	Methyldopa	UK	750–2000 mg/day	Oral	Hypertension	9	28	33.9 (mean)	Other***
[54]	**Schneider H et al. (1988)**	Labetalol	Switzerland	Ex vivo	Ex vivo	Ex vivo	5	Ex vivo 5 studysize*2 maternal and fetal*12 time points = 120	Unknown (healthy placentas from term deliveries)	Other***
[53]	**Nandakumaran M et al. (1989)**	Labetalol	Kuwait	Ex vivo	Ex vivo	Ex vivo	6	Ex vivo 6 study size*2 maternal and fetal*6 time points = 72	Unknown (healthy placentas from term deliveries)	Other***
[47]	**Rubin PC et al. (1983)**	Labetalol	Scotland	50 mg single dose	Intravenous	Hypertension	20	20 (subjects) * 15 (time points) = 300	33–38 (range)	popPK
[55]	**Nylund L et al. (1984)**	Labetalol	Sweden & Kuwait	200 mg/6 h	Oral	Hypertension	7	22	Unknown	Other***
[49]	**Lunell NO et al. (1985)**	Labetalol	Sweden	600––1200 mg/day	Oral	Gestational hypertension	8	32	34.4 ± 12.4 (mean ± SD)	Other***
[48]	**Rogers RC et al. (1990)**	Labetalol	USA	100 mg/8 h	Oral	Gestational hypertension	8	Unknown	Unknown	NCA
[46]	**Saotome et al. (1993)**	Labetalol	Japan	150–450 mg/12 h	Oral	Hypertension	7	7*7 = 49	34–37 (range)	NCA
[51]	**Boulton DW et al. (1999)**	Labetalol	USA	100 mg single dose	Oral	Hypertension	4	4 (subjects) * 2(maternal andumbilical) = 8	37–40 (range)	Other***
[52]	**Carvalho et al. (2009)**	Labetalol	Brazil	100 mg single dose	Oral	Hypertension	1	Human 13	Unknown	popPK
[45]	**Carvalho et al. (2011)**	Labetalol	Brazil	100 mg vs. 40 mg	Oral vs. intravenous	Hypertension in patients with diabetes	30	30 (subjects * 17 (time points iv) + 30 * 16 (time points oral administration)= 990	28.9–40.0 (range)	popPK
[42]	**Uematsu et al. (2013)**	Labetalol	Japan	50–100 mg/8 h	Oral	Gestational hypertension	29	58	36.3 ± 3.5 (mean ± SD)	popPK
[50]	**Fischer et al. (2014)**	Labetalol	USA	50–2400 mg/day	Oral	Chronic/gestational hypertension	57	649	20 (11–39) (mean (range))	popPK
[62]	**Poranen AK et al. (1998)**	Nifedipine	Finland	Ex vivo	Ex vivo	Ex vivo	5	Ex vivo 5*16 = 80	40.4 (39–42) (mean (range))	NCA
[57]	**Pirhonen JP et al. (1990)**	Nifedipine	Finland	Single dose of 20 mg	Oral	Normotensive	10	10 (subjects) * 5 (time points) = 50	38.8 (37–39) (mean ((range)	Other***
[61]	**Manninen AK et al. (1991)**	Nifedipine	Finland	10 mg/8 h	Oral	Hypertension	11	66	36 (31–39) (mean ((range)	Other***
[65]	**Prevost RR et al. (1992)**	Nifedipine	USA	10 mg/6 h	Oral	Gestational hypertension	15	approx. 15*15 = 225	32.1 ± 2.7 (mean ± SD)	NCA
[66]	**Filgueira et al. (2015)**	Nifedipine	Brazil	20 mg/12 h	Oral (slow-release)	Hypertension	12	approx. 12*30 = 360	36.1 ± 1.7 (mean ± SD)	NCA
[39]	**Filgueira et al. (2017)**	Nifedipine	Brazil	20 mg/12 h	Oral (slow-release)	Hypertension	22	22*23 = 506	Control 39.1 (38.7–39.5); case (*N* = 10) 36.9 (35.2–38.4) (mean ((range)	NCA
[63]	**Ferguson IJE et al. (1989)**	Nifedipine	USA	20 mg/6 h	Oral and sublingual	Preterm labor	13	9 time points at every study day per subjectt	Unknown	Other***
[67]	**Marin TZ et al. (2007)**	Nifedipine	Switzerland	4 × 10 mg* followed by 1 × 60 mg	Oral (capsules and continuous release)	Preterm labor	24	24*1 = 24	22–34 (range)	Other***
[58]	**Papatsonis DNM et al. (2007)**	Nifedipine	Netherlands	4 × 10 mg* followed by 1 × 20 mg	Oral (capsules and slow-release tablet)	Preterm labor	5	5 (subjects) * 18 (time points) = 140	31.3 ± 0.9 (30–33 4/7) (mean ± SD (range))	Other***
[59]	**Silberschmidt AL et al. (2008)**	Nifedipine	Switzerland	30–150 mg/day	Oral	Preterm labor	40	40 (subjects) * 5 (time points) = 200	34.7 ± 0.8 (mean ± SD)	Other***
[60]	**Haas DM et al. (2012)**	Nifedipine	USA	4 × 10 mg* and 2 × 20 mg	Oral (capsules and slow-release tablet)	Preterm labor	20	20 (subjects) * 8 (time points) = 160	29.7 ± 2.7 (24–34) (mean ± SD (range))	NCA
[64]	**Haas et al. (2013)**	Nifedipine	USA	At SS: 10–20 mg/6–8 h	Oral (immediate-release)	Preterm labor	14	9 time points at every study day per patient	32 ± 4 (24–36) (mean ± SD (range))	NCA
[56]	**Ter Laak et al. (2015)**	Nifedipine	Netherlands	20 mg/6 h vs. placebo	Oral (slow-release tablet)	Preterm labor	11	11 (subjects) * 7 (time points) = 77	26–32 2/7 (range)	popPK
[71]	**Ke AB et al. (2012)**	Nifedipine	USA	10 mg/6 h	Oral	Gestational hypertension	**	PBPK model	Unknown	PBPK
[69]	**Quinney et al. (2012)**	Nifedipine	USA	At SS: 10–20 mg/6–8 h	Oral	Preterm labor	**	PBPK model	Unknown	PBPK
[68]	**Dallmann A et al. (2018)**	Nifedipine	Germany	NA	Oral	Unknown	**	PBPK model	Unknown	PBPK

*Ref* reference number, *UK* United Kingdom, *USA* United States of America, *h* hours, *SS* steady state, *g* gram, *mg* milligram, *vs.* versus, *T* time, *kg* kilograms, *PBPK* physiologically based pharmacokinetics, *popPK* population pharmacokinetics, *NCA* noncompartmental analysis

^*^T = 0, 15, 30, 45 min; **No new patient data is generated; ***Only concentrations reported

**Table 2:** Results of fetal (cord) plasma/maternal plasma and amniotic fluid/maternal plasma ratios of methyldopa

	***N***	**Fetal (cord) plasma/maternal plasma**			**Amniotic fluid/maternal plasma**			**Dosage**	**Gestational age**
		**Free**	**Sulfate conjugated**	**Total**	**Free**	**Sulfate conjugated**	**Total**		
Jones et al. [43]	12	1.19 (*N* = 12)	0.79 (*N* = 12)	0.99 (*N* = 12)	1.0 (*N* = 6)	2.0 (*N* = 6)	1.23 (*N* = 6)	750–2000 mg/day	Unknown
Jones et al. [44]	9	0.93 (*N* = 4)	1.49 (*N* = 4)	0.63 (*N* = 5)	2.04 (*N* = 2)	6.67 (*N* = 2)	3.85 (*N* = 2)	750–2000 mg/day	33.9 (mean)

Values are reported as mean ratios. In original articles, maternal plasma/fetal (cord) plasma and maternal plasma/amniotic fluid ratios were given; for consistency, this was converted to fetal (cord) plasma/maternal plasma and amniotic fluid/maternal plasma ratios

Jones HM et al. [43]. Total amniotic fluid concentrations ranged from 200 to 3600 ng/mL with a dose of 250 mg/8 h–500/6 h at delivery, 4–12 h after the last dose of methyldopa

Jones HM et al. [44]: Total amniotic fluid concentrations ranged from 1580 to 2520 ng/mL with a dose of 500 mg/12 h–500/6 h at delivery, 4–28 h after the last dose of methyldopa

**Table 3:** Results of pharmacokinetic parameters of labetalol

**Pharmacokinetic parameter (unit)**	**Rogers et al. **[48]	**Saotome et al. **[46]
*N*	8	7
Dosage	100 mg/8 h	150–450 mg/12 h
Elimination half-life (hours)	1.7 ± 0.27	5.8 ± 0.3 (4.3–6.9*)
Clearance (mL/kg/min)	21.8 ± 6.8	43.7 ± 5.2 (31.9–73.3*)
Time of maximum serum concentration (minutes)	20	NA
T_max_ with food ingestion (minutes)	60	Approximately 60

Values are mean ± standard deviation

^*^is a range

**Table 4:** Results of fetal/maternal plasma ratios of nifedipine

**Study**	***N***	**Indication**	**F/M ratio**	**A/M ratio**	**Dose**	**Gestational age**
**Poranen et al. **[62]	5	Ex vivo (placental transfer)	0.054 ± 0.020	NA	Ex vivo	40.4 (39–42) (mean (range))
**Pirhonen et al. **[57]	10	Normotensive/research	0.76	NA	Single dose of 20 mg	38.8 (37–39) (mean ((range)
**Manninen et al. **[61]	11	Hypertension	0.8 ± 0.1	0.3 ± 0.1	10 mg/8 h	36 (31–39) (mean ((range)
**Prevost et al. **[65]	15	Pregnancy-induced hypertension	0.93 ± 0.2	0.56 ± 0.1	10 mg/6 h	32.1 ± 2.7 (mean ± SD)
**Filgueira et al. **[66]	12	Hypertension	NA	0.05 (0.03–0.06)	20 mg/12 h	36.1 ± 1.7 (mean ± SD)
**Filgueira et al. **[39]	22 (12 controls vs. 10 T2DM)	Hypertension	0.53 (T2DM 0.44)	0.05 (T2DM 0.05)	20 mg/12 h	control 39.1 (38.7–39.5); case (*N* = 10) 36.9 (35.2–38.4) (mean ((range)
**Silberschmidt et al. **[59]	40	Preterm labor	0.77	NA	30–150 mg/day	34.7 ± 0.8 (mean ± SD)

Values are mean ± standard deviation

*T2DM* type 2 diabetes mellitus, *F/M ratio* umbilical serum/maternal serum ratio, *A/M ratio* amniotic fluid serum/maternal serum ratio, *NA* not applicable

**Table 5:** Pharmacokinetic parameters of nifedipine as tocolytic

**Study**	**Ferguson et al. **[63]	**Marin et al. **[67]	**Papatsonis et al. **[58]	**Silberschmidt et al. **[59]	**Haas et al. **[60]	**Haas et al. **[64]	**Ter Laak et al. **[56]
Oral dose	10–40 mg in first hour followed by 20 mg/4–6 h (sublingual)	* followed by 60 mg continuous release nifedipine	* followed by 20 mg slow release (T = 105 min)	30–150 mg/day tablets with sustained release	* and 1 × 20 mg (T = 105 min)	10–20 mg/6–8 h	20 mg/6 h vs. placebo
Elimination half-life (t_1/2_)	81 min (49–137)	NA	NA	maternal 17.4 h (95% CI: 13.9–21.7); fetal (umbilical cord) 20.4 h (95% CI: 15.7–26.3)	NA	1.68 ± 1.56 h	2–5 h
Volume of distribution (V_d_)	NA	NA	NA	NA	NA	NA	6.2 ± 1.9 L/kg
Time of maximum serum concentration (T_max_)	NA	NA	1.2 ± 0.1 h	`NA	1 h	NA	NA
Maximum serum concentration	96.7 ± 45.3 (23.4–197.9) ng/mL	NA	127 ± 44	NA	NA	NA	NA
Area under the plasma drug concentration–time curve (AUC)	NA	NA	NA	NA	Mean 86.1 ± 61.1 µg*h/L	AU_C0-6 h_: 207 ± 138 µg*h/L	NA
Maternal mean nifedipine concentration	7.2 ± 5.5 ng/mL after 20 mg nifedipine orally every 6 h	32.9 ± 25.1 ng/mL (6–101 ng/mL)	67.4 ± 28.4 ng/mL	NA	NA	NA	16.8 ng/mL (median concentration at SS)
Neonatal serum plasma concentration after delivery	1.8–29.5 ng/mL (*N* = 5)	NA	NA	NA	NA	NA	NA

Given means are reported ± SD unless stated otherwise

*NA* not applicable, *h* hours

^*^4 × 10 mg nifedipine (T = 0, 15, 30, 45 min)

The most important reported ex vivo (placental perfusion model) and in vivo (absorption, distribution, metabolism, excretion, and fetal (cord blood)/maternal ratio data are described below for all drugs).

### Methyldopa

#### In vivo

Only two studies by the same group reported on the PK during pregnancy (delivery) and placental transfer of methyldopa (Table 2) [43, 44]. The first study described the free, sulfated, and total methyldopa concentration of 12 pregnant women in maternal plasma at delivery (*N* = 12), fetal (umbilical cord) plasma (*N* = 12), and amniotic fluid (*N* = 6) [43]. Methyldopa undergoes conjugation with sulfate to improve renal excretion of the drug. Total maternal plasma concentrations ranged between 200 and 2400 ng/mL and fetal (umbilical cord) plasma concentrations ranged between 250 and 2700 ng/mL following a dose ranging from 250 mg/8 h to 500 mg/6 h and a time of delivery of 2–16 h after the last dose of methyldopa. The fetal/maternal plasma ratio of methyldopa (*N* = 12) was 1.19 (free), 0.79 (sulfate conjugated), and 0.99 (total). The calculated ratios showed that both free and conjugated methyldopa concentrations were similar in maternal and fetal (cord) plasma. The amniotic fluid/maternal plasma ratio of methyldopa (*N* = 6) was 0.77 (free), 2.0 (conjugated), and 1.23 (total). Overall, the total methyldopa concentration in the amniotic fluid was 19% higher than the maternal plasma concentrations. The conjugated concentration in amniotic fluid was on average two times higher than the conjugated concentration in maternal plasma and in five out of six cases noticeable higher than the free form in amniotic fluid.

The second study described, similar to the previous study, the concentration of free, sulfate conjugated and total methyldopa in maternal plasma (*N* = 5), fetal (umbilical cord) plasma (*N* = 7), amniotic fluid (*N* = 4), and neonates (*N* = 7) [44]. Total maternal plasma ranged from 422 to 3543 ng/mL and fetal (umbilical cord) plasma from 154 to 1802 ng/mL with a dose range of 250 mg/6 h–500 mg/6 h and a time of delivery after the last dose of methyldopa of 4–14 h. Total fetal/maternal (cord) plasma ratio was 0.9 (free; *N* = 4), 0.5 (conjugated; *N* = 4), and 0.6 (total; *N* = 5). Total amniotic fluid concentrations ranged from 1580 to 2520 ng/mL with a dose of 500 mg/12 h–500 mg/6 h and a time of delivery after the last dose of methyldopa of 4–28 h. Amniotic fluid/maternal plasma ratio of methyldopa was 2.0 (free; *N* = 2), 6.7 (conjugated; *N* = 2), and 3.8 (total; *N* = 2). Both studies show disparities, for instance, for the maternal plasma to fetal plasma for sulfate conjugated methyldopa ratio (0.79 vs. 1.49). The authors do not give an explanation for these differences between their studies, although they speculate whether these higher conjugated drug levels are due to fetal conjugation or to placental transfer, which might also vary between fetuses.

### Labetalol

Eleven studies reported on the PK of labetalol during pregnancy and its placental transfer [42, 45–55].

#### Ex vivo (placental transfer)

Two out of the 11 studies on labetalol were ex vivo studies using isolated human cotyledons from normal pregnancies with term deliveries to investigate the placental transfer of labetalol [53, 54]. Both studies showed placental transfer of labetalol and placental tissue binding. Schneider et al. (*N* = 5) reported a placental transfer at steady state of 16.6% ± 4.6% [54]. After bolus injection, placental transfer of labetalol was clearly suppressed, as a result of high placental tissue binding [54]. Different albumin levels (0.02 and 4.0 g/dl) had little effect on the labetalol transfer [54]. Nandakumaran et al. (*N* = 6) showed a ratio of labetalol transfer of 5% ± 0.7% and compared to antipyrine 32% ± 2.8% at steady-state perfusion conditions [53].

#### In vivo

The reported studies on the PK during pregnancy and placental transfer of labetalol used different outcome measures for PK, which makes a clear overview difficult. Studies assessed absorption, distribution, metabolism, and excretion in pregnant women. The largest studies are described below.

Rubin et al. investigated the PK of labetalol in ten hypertensive pregnant women before and after delivery and ten normotensive women [47]. V_d_ and CL were not significantly different in these three groups [47]. Nylund et al. determined labetalol plasma levels in seven pregnant women [55]. Measured plasma levels in pregnant women were generally lower compared with nonpregnant women with a similar dose in earlier studies. The fetal/maternal labetalol concentration ratio was about 0.5 in four out of five investigated patients and in the other patient about 1 [55]. A similar ratio (0.67) was found in a Japanese study in 29 patients [42]. Lunell et al. studied transfer of labetalol into the amniotic fluid [49]. In six of eight patients, the labetalol concentration in amniotic fluid was lower than that in maternal plasma.

Rogers et al. and Saotome et al. determined PK parameters of labetalol in pregnant women with different results for elimination half-life and clearance (Table 3) [46, 48]. For instance, elimination half-life ranged from 1.7 to 5.8 h; even while quite different, they are both short implicating that dosing three times daily is optimal. The umbilical cord fetal/maternal serum ratio was 0.5 ± 0.15 and amniotic fluid/maternal serum ratio 0.16 ± 0.13 in Rogers et al.; Saotome et al. mainly focused on the relation between labetalol levels and blood pressure [46, 48]. Boulten et al. and Carvalho et al. studied the transplacental distribution of labetalol stereoisomers during pregnancy and at delivery [51, 52]. The pharmacological active stereoisomers (RR and SR) had a lower plasma concentration compared to the inactive stereoisomers (SS and RS) [52]. The ratio AUC_(RR)_/AUC_(SS)_ was 0.5 [52]. The inactive stereoisomer, SS, was present in the highest concentration in both maternal plasma and fetal (cord) plasma in all four subjects when labetalol was detectable [51]. The effect of gestational diabetes mellitus on the stereoselective kinetic disposition and metabolism was assessed by Carvalho et al. because diabetes mellitus can alter enantioselective PK processes potentially leading to a more outspoken blood pressure decrease in patients with diabetes [45]. Indeed, the AUC of the SS and SR isomers were higher in the diabetic women compared to the nondiabetic after oral administration [45]. The PK of labetalol was not stereoselective after intravenous administration [45]. Fischer et al. studied the influence of gestational age and body weight on the PK of labetalol in pregnancy [50]. Oral clearance (CL/F) ranged from 1.4-fold higher at 12 weeks gestational age and 1.6-fold higher at 40 weeks compared to postpartum (up to 12 weeks after delivery) CL/F; data was collected within the same patient [50]. The apparent V_d_ of the central compartment during pregnancy was 1.9-fold higher [50]. Plasma proteins concentrations, alpha-1 acid glycoprotein and albumin, were lower during pregnancy compared postpartum as both are decreased during pregnancy, although not significant in this cohort [50].

### Nifedipine

Sixteen studies reported on nifedipine PK in pregnant women [38, 39, 56–69]. Six out of these 16 studies reported on the placental transfer investigating the fetal/maternal ratio of nifedipine plasma concentrations (Tables 4 and 5) [39, 59, 61, 62, 65, 66]

#### Ex vivo (placental transfer)

One out of the 16 studies on nifedipine was an ex vivo study published in 1998 by Poranen et al. using five isolated human placental cotyledons (Table 4) [62]. The mean ± SD placental CL of nifedipine at steady state was 0.54 ± 0.20 mL/min [62]. Placental transfer of nifedipine was 5.4% ± 2.0% and the CL index (the ratio between nifedipine and the internal standard antipyrine) 0.41 [62]. The recovery of the added nifedipine in the perfusion buffer was 51% ± 9.3%, although nifedipine bound to placental tissue was not measured [62].

#### In vivo

##### Normotensive

One out of the 16 studies on nifedipine investigated the PK of ten normotensive healthy pregnant women after a single oral dose of 20 mg nifedipine [57]. The mean maternal serum concentration of nifedipine before birth was 38.3 ± 26.6 ng/mL (70–90 min after intake) and at birth (on average 165 min (range 150–180 min) after intake) 17.6 ± 12.7 ng/mL [57]. The mean concentration in the umbilical vein (afferent) was 13.1 ± 14.0 ng/mL and in the umbilical artery (efferent) 10.0 ± 9.4 ng/mL [57]. Umbilical venous fetal/maternal ratio of nifedipine 2–3 h after nifedipine intake was 0.76 [57]. After one blood circulation of the drug in the fetus, approximately 25% of nifedipine had distributed in the fetal tissues [57].

##### Hypertension

Five out of the 16 studies on nifedipine studied the PK in hypertensive pregnant women [39, 61, 65, 66], of which one developed a PBPK model as described below [70].

Manninen et al. studied nifedipine concentrations in maternal and umbilical fetal serum, amniotic fluid, breast milk, and urine of mothers and offspring (Table 4) [61]. The mean (± SD) serum concentration in 11 third trimester pregnant women (4.3 ± 3.1 ng/mL) was lower than that in 6 different nonpregnant controls (12.0 ± 2.9 ng/mL) with the same dose of nifedipine (10 mg three times daily) [61].

The fetal/maternal serum ratio was 0.8 ± 0.1 and the amniotic fluid serum/maternal serum ratio 0.3 ± 0.1 at delivery [61].

Prevost et al. investigated the disposition of nifedipine in pregnant women using nifedipine 10 mg every 6 h (mean gestation 32.1 weeks; Table 4) [65]. At steady state, the C_max_ was 38.6 ± 18 ng/mL 40 min after ingestion, t_1/2_ 1.3 ± 0.5 h, mean CL/F 2.0 ± 0.8 L/h/kg, and the AUC 83.2 ± 42.6 ng*h/mL [65]. The umbilical cord fetal/maternal serum ratio was 0.93 ± 0.2 and the amniotic fluid/maternal serum ratio 0.56 ± 0.15 [65]. Filgueira et al. reported the following PK parameters in pregnant women using nifedipine 20 mg every 12 h: AUC_0-12_ 250 ng*h/mL, CL_t_/F 89.2 L/h, V_d_/F 600 L, and t_1/2_ 5.1 h (Table 4) [66]. The amniotic fluid/plasma concentration ratio was very low: on average 0.05 ranging from 0.03 to 0.06 based on AUC^0−12^ [66]. In a later study, the same research group tested the effect of type 2 diabetes mellitus (T2DM) on the PK and transplacental transfer of nifedipine in hypertensive pregnant women using the same dosing regimen (Table 4) [39]. There was no effect of T2DM on the PK or placental transfer of nifedipine [39].

##### Tocolysis

Eight out of the 16 studies on nifedipine studied the PK of pregnant women undergoing tocolysis [56, 58–60, 63, 64, 67, 69] (Table 5) of which one developed a PBPK model as described below [69].

In a study by Ferguson et al*.*, PK parameters of nifedipine for tocolysis were measured after sublingual administration and oral administration (Table 5) [63]. Nifedipine plasma concentrations were also measured in 11 neonates at delivery after multiple doses. In six of them, the nifedipine concentration was undetectable (below LLQ) and in the other five newborns values ranged from 1.8 to 29.5 ng/mL [63]. Results of comparable studies of Marin et al. and Papatsonis et al. can be found in Table 5 [58, 67]. For nonpregnant women, a V_d_ and t_1/2_ have been described in the literature, 1.2 ± 1.3 L/kg [58] and 6–11 h, respectively. Silberschmidt et al. determined the nifedipine concentration and other PK parameters (Table 5) in maternal and fetal blood after tocolysis with gastrointestinal therapeutic system (GITS) tablets, a modified release formulation with sustained release. The mean fetal plasma/maternal plasma ratio was 0.77 [59]. The linear regression between maternal and fetal concentrations was significant [59]. Haas et al. performed a pilot study about the impact of genotype on PK of nifedipine indicated for tocolysis; results are shown in Table 5 [60]. The nifedipine/oxidized nifedipine (nif/ox) AUC ratio was 2.83 ± 4.20 [60]. Expression of CYP3A5 (defined as at least one CYP3A5*1 allele, 5/20 subjects) did have a statistically significant effect on nifedipine exposure (expresser (exp): 139.5 ± 97.3 ng/mL/h vs. nonexpressers (non): 68.3 ± 31.8 ng/mL/h, *p* = 0.02) and the nif/ox ratio (exp: 6.33 ± 7.82 vs. non: 1.67 ± 0.834, *p* = 0.03) [60]. Four subjects (2 exp and 2 non) used CYP3A inhibiting co-medication and had significantly higher nifedipine exposure (*p* < 0.0001) independent of CYP3A5 genotype [60]. These results were confirmed in a later study (Table 5) [64]. The CL/F was significantly different (*p* = 0.007) between high and low expressers of CYP3A5 (232.0 ± 37.8 µg/mL vs. 85.6 ± 45 µg/mL, respectively) [64]. Furthermore, the average nifedipine plasma concentration, Cl/F, and V_d_/F of the high and low expressers were significantly different [64]. Ter Laak et al. described the PK (t_1/2_, V_d_, and median nifedipine concentration at steady state) of maintenance slow-release nifedipine as tocolytic (Table 5) [56].

##### Physiologically based pharmacokinetics (PBPK)

Three out of the 16 studies on nifedipine developed a PBPK model [38, 68, 71]. Ke et al. [71] developed a PBPK model based on third trimester data [65]. The mean CL/F of nifedipine at steady state was almost doubled during pregnancy (145.7 L/h vs. 74.4 L/h) [71], which may have implications for the pharmacodynamic effects of nifedipine in pregnant women if the same dosing is applied. The PBPK model predicted the following nonpregnant vs. third trimester pregnant data of nifedipine (observed ratio): mean steady state AUC ratio of 2.1 (2.0), C_max_ 2.1 (1.8), and C_min_ 2.4 (3.1) [71]. Quinney et al. performed a semi-mechanistic metabolism model of midazolam and nifedipine (based on Haas et al. CYP3A substrates, in obstetric patients) [38, 64]. Nifedipine steady-state AUC predicted by the developed model was underestimated by 11% (210 (121–299) ng*h/mL vs. 237 (224–253) ng*h/mL) and the C_max_ was overestimated by 3% (178 (166–188) ng/mL vs. 184 (90–308) ng/mL) [38]. The third PBPK model was developed by Dallmann et al. to predict the PK during pregnancy of drugs metabolized via several enzymatic pathways [68]. The C_max_ of both pregnant and nonpregnant women was underestimated by their model [68]. Ninety-three percent of the predicted mean plasma concentrations of nifedipine in pregnant women fell within the twofold error range and 54% within the 1.3-fold error rate.

## Discussion and conclusion

This is the first systematic review that provides a complete overview of what is currently known on PK of the most commonly used antihypertensive drugs throughout pregnancy: methyldopa, labetalol, and nifedipine. Since variation of the fetal/maternal ratio of methyldopa, labetalol, and nifedipine is expected to be high, values < 0.1 (limited transfer), 0.1–1 (transfer), and > 1 (fetal accumulation) were considered concordant. All the identified ratios were between 0.1 and 1 which means that there is placental transfer of the three investigated drugs, but no fetal accumulation. Identified parameters for the same drug could vary substantially between studies. We assume that the small sample sizes and the challenges of sampling before, during, and after delivery including amniotic fluid may have led to uncertainties and discrepancies, including the fact that often point measurements instead of preferable AUC estimates were available.

Especially studies on methyldopa are scarce, while this is still considered first-line treatment of hypertension during pregnancy. For labetalol and nifedipine, the reports were highly variable in the investigated dosages, indications, patients, PK estimates, and study designs (ex vivo and in vivo). Furthermore, the studies differed in quality, methods to describe the PK, patient size, the number of samples, and the timing of sampling in relation to the dose as well as the gestational age. A few studies also described the concentration of the drug in amniotic fluid, for example, in relation to maternal plasma which might lead to prolonged exposure. The number of studies was low and data was mostly not homogeneous, often lacking ratios between maternal and fetal plasma concentrations, which made it difficult to systematically present data or perform meta-analyses. Relevant differences in PK parameters of the same drug were reported; however, it should be considered that PK parameters such as AUC and C_max_ are dose dependent. Despite the frequent use of these three drugs during pregnancy according to international and national protocols, appropriate descriptions of the impact of pregnancy on the population PK are still sparse and inconsistent, while indispensable to truly assess the pharmacodynamics of these drugs during pregnancy.

Although methyldopa is first-choice drug for hypertensive disorders in pregnant women in many countries, only two studies reported on methyldopa PK in pregnant women. The free fetal/maternal ratio of methyldopa was as expected in the range of 1 [43, 44]. In amniotic fluid, the concentration of conjugated, i.e., inactive, methyldopa was higher than that of the free form. This can be explained by either less reabsorption of the conjugated form by the fetus or more renal excretion of the free form by the fetus. Both free and conjugated methyldopa were lower in arterial umbilical cord (fetal) plasma compared to venous umbilical cord (fetal) plasma which indicated that the fetus eliminates both forms and is actively involved in methyldopa disposition.

Three studies reported on in vivo placental transfer of labetalol [46, 48, 55]. Fetal/maternal serum ratios were about 0.5 with one unexplained outlier of about 1. Labetalol is a lipid-soluble drug which makes it easier to pass the placenta compared to a hydrophilic component by passive diffusion. Furthermore, there is a large unexplainable difference in the reported clearance values of labetalol [46, 48]. Studies that also addressed the ratio between active and inactive stereoisomers concluded that inactive isoforms seem to predominate both in maternal and in fetal samples.

Five studies reported on the umbilical fetal/maternal serum ratio of nifedipine [39, 57, 59, 61]. Ratios ranged from 0.53 to 0.93, meaning there is placental transfer of nifedipine but no fetal accumulation. This was confirmed in an ex vivo placental transfer study, although the ratio was even lower here (0.054). Two studies by Haas et al. showed that CYP3A5 genotype influences the nifedipine concentrations when used as tocolytic [60, 64]. Due to these PK changes during pregnancy and placental differences per trimester, it can be clinically relevant to adjust dose and dosing interval of nifedipine for the different indications during pregnancy, especially in low expressors of CYP3A5 or differences activity of CYP3A4 due to pharmacogenetic variation or interactions (not specifically studied) [61].

More detailed knowledge about the PK of methyldopa, nifedipine, and labetalol may lead to better understanding of the impact on the PD including safety consequences for the developing fetus and the offspring during the life course. A fetus is assisted by the mother to clear the drug during intrauterine exposure by placental transfer back to the mother. From birth onwards, the newborn is fully responsible for the clearance, despite its still immature drug clearance capacity. This applies even more to preterm infants, while preterm birth is commonly seen after the maternal indications for which methyldopa, labetalol, and nifedipine were prescribed. Their drug clearance capacity is even more limited.

This review serves as a good basis to identify the need for future research with the ultimate goal to reach evidence-based dosing for this vulnerable population, including the developing placenta and fetus. Currently, dosing is empirical and based on PD of the mother, since our knowledge or understanding of PD or PK is too limited to guide prescription in pregnancy. Although major adverse events for the described drugs are scarce, combining PK and PD, required drug dosages might be further minimized, hereby potentially lowering exposure of the fetus and the newborn.

As a start towards evidence-based personalized dosing in the future, further studies combining therapeutic drug monitoring (PK) studies designed with contemporary approaches to get more homogenous data and PD by ways of monitoring hemodynamics and other health parameters of pregnant women including the breastfeeding phase and their offspring are needed [72]. For example, PBPK modeling is a promising approach to evaluate different dosing regimens in pregnant women, because it can predict the influence of the physiological changes in the body [15, 73]. From these data, personalized dosing based on maternal and pregnancy characteristics, if necessary combined with therapeutic drug monitoring, can be optimized. Evidence-based personalized dosing in the future can mean that dosing will be based on trimester, weight, and/or individual characteristics (like disease, genetic polymorphism) throughout gestation.

In general, including, instead of excluding, pregnant women in clinical trials on drugs commonly required during pregnancy would give the best information. More use of standardized ex vivo placental transfer models of different gestational ages (1^st^, 2^nd^, and 3^rd^ trimester) or “placenta-on-a-chip” before clinical trials would make this safe and helps in dose finding [74].

## Supplementary Information

Below is the link to the electronic supplementary material.

Supplementary file1 (DOCX 21 KB)

## Data Availability

Data sharing not applicable to this article as no datasets were generated or analyzed during the current study. All data given are available in the original articles.
