# Supplementary material for: Pharmacokinetics of the most commonly used antihypertensive drugs throughout pregnancy methyldopa, labetalol, and nifedipine: a systematic review
Source: Eur J Clin Pharmacol. 2022 Sep 15;78(11):1763–76. doi: 10.1007/s00228-022-03382-3 (PMC9474278; doi:10.1007/s00228-022-03382-3)
Supplement: Supplementary file 1 — Supplementary file1 (DOCX 21 KB) [file 228_2022_3382_MOESM1_ESM.docx]

**Supplementary materials**

**Appendix 1: Literature search**

**Embase – 966 refs**

('labetalol'/exp OR 'nifedipine'/exp OR 'methyldopa'/exp OR (labetalol* OR labetolol* OR trandate* OR nifedipin* OR adalat* OR methyldopa* OR aldomet*):ab,ti) **AND** ('pharmacokinetics'/exp OR pharmacokinetics:lnk OR (pharmacokinetic* OR kinetic* OR accumulat* OR diffus* OR disposit* OR distribut* OR elimin* OR excret* OR half-life* OR penetrat* OR area-under* OR concentrat* OR auc):ab,ti) **AND** ('pregnancy disorder'/exp OR 'pregnant woman'/exp OR 'pregnancy'/exp OR 'fetus'/exp OR 'placenta'/exp OR 'placental transfer'/exp OR 'fetomaternal transfusion'/exp OR (prematur* OR immatur OR prenatal* OR perinatal* OR umbilical-cord* OR embryo* OR frigid* OR pre-eclamp* OR preeclamp* OR eclamp* OR maternal* OR pregnan* OR fetus* OR gestat* OR gravidit* OR childbear* OR labor OR labour* OR child*-bear* OR materno* OR fetal* OR feto* OR foetal* OR foetus* OR diaplacenta* OR paraplacenta* OR transplacenta* OR placenta*):ab,ti) *NOT ([animals]/lim NOT [humans]/lim) AND [english]/lim NOT ([Conference Abstract]/lim AND [1800-2016]/py)*

**Medline – 318 refs**

(exp Labetalol/ OR exp Nifedipine/ OR exp Methyldopa/ OR (labetalol* OR labetolol* OR trandate* OR nifedipin* OR adalat* OR methyldopa* OR aldomet*).ab,ti.) **AND** (exp Pharmacokinetics/ OR pharmacokinetic*.fs. OR (pharmacokinetic* OR kinetic* OR accumulat* OR diffus* OR disposit* OR distribut* OR elimin* OR excret* OR half-life* OR penetrat* OR area-under* OR concentrat* OR auc).ab,ti.) **AND** (exp Pregnancy Complications/ OR exp Pregnancy/ OR exp Pregnant Women/ OR exp Fetus/ OR exp Placenta/ OR exp Fetomaternal Transfusion/ OR (prematur* OR immatur OR prenatal* OR perinatal* OR umbilical-cord* OR embryo* OR frigid* OR pre-eclamp* OR preeclamp* OR eclamp* OR maternal* OR pregnan* OR fetus* OR gestat* OR gravidit* OR childbear* OR labor OR labour* OR child*-bear* OR materno* OR fetal* OR feto* OR foetal* OR foetus* OR diaplacenta* OR paraplacenta* OR transplacenta* OR placent*).ab,ti.) *NOT (exp animals/ NOT humans/) AND english.la.* NOT (news OR congres* OR abstract* OR book* OR chapter* OR dissertation abstract*).pt.

**Cochrane (RCTs) – 24 refs**

((labetalol* OR labetolol* OR trandate* OR nifedipin* OR adalat* OR methyldopa* OR aldomet*):ab,ti) **AND** ((pharmacokinetic* OR kinetic* OR accumulat* OR diffus* OR disposit* OR distribut* OR elimin* OR excret* OR (half NEXT/1 life*) OR penetrat* OR (area NEXT/1 under*) OR concentrat* OR auc):ab,ti) **AND** ((prematur* OR immatur OR prenatal* OR perinatal* OR (umbilical NEXT/1 cord*) OR embryo* OR frigid* OR (pre NEXT/1 eclamp*) OR preeclamp* OR eclamp* OR maternal* OR pregnan* OR fetus* OR gestat* OR gravidit* OR childbear* OR labor OR labour* OR (child* NEXT/1 bear*) OR materno* OR fetal* OR feto* OR foetal* OR foetus* OR diaplacenta* OR paraplacenta* OR transplacenta* OR placenta*):ab,ti)

**Web of Science – 298 refs**

TS=(((labetalol* OR labetolol* OR trandate* OR nifedipin* OR adalat* OR methyldopa* OR aldomet*)) **AND** ((pharmacokinetic* OR kinetic* OR accumulat* OR diffus* OR disposit* OR distribut* OR elimin* OR excret* OR half-life* OR penetrat* OR area-under* OR concentrat* OR auc)) **AND** ((prematur* OR immatur OR prenatal* OR perinatal* OR umbilical-cord* OR embryo* OR frigid* OR pre-eclamp* OR preeclamp* OR eclamp* OR maternal* OR pregnan* OR fetus* OR gestat* OR gravidit* OR childbear* OR labor OR labour* OR child*-bear* OR materno* OR fetal* OR feto* OR foetal* OR foetus* OR diaplacenta* OR paraplacenta* OR transplacenta* OR placenta*)) NOT ((animal* OR rat OR rats OR mouse OR mice OR murine OR dog OR dogs OR canine OR cat OR cats OR feline OR rabbit OR cow OR cows OR bovine OR rodent* OR sheep OR ovine OR pig OR swine OR porcine OR veterinar* OR chick* OR zebrafish* OR baboon* OR nonhuman* OR primate* OR cattle* OR goose OR geese OR duck OR macaque* OR avian* OR bird* OR fish*) NOT (human* OR patient* OR women OR woman OR men OR man))) AND DT=(Article OR Review) AND LA=(English)

**Google Scholar – random top 200 refs**

labetalol|labetolol|trandate|nifedipine|adalat|methyldopa|aldomet pharmacokinetics|kinetics| pharmacokinetic|kinetic premature|immature|prenatal|perinatal|embryo|frigid|preeclampsia|eclampsia|maternal|pregnancy|pregnant|fetus|gestation|gravidity|childbear|labor|labour|materno|fetal|feto|foetal|foetus|diaplacenta|paraplacenta|transplacenta|placenta

**Supplementary Table 1. Pharmacokinetic properties of methyldopa, labetalol and nifedipine (information from Summaries of Product Characteristics and references as indicated in the table; dosages in pregnancies based on Clinical Practicy by Seely et al.[75])**

| **Drug** | **Mechanism of action** | **Main indication** | **Metabolism** | **Dosages used in pregnancy (per os)** | **Therapeutic range in non-pregnancy persons** | **Main adverse effects** |
| --- | --- | --- | --- | --- | --- | --- |
| Methyldopa | centrally acting sympathomimetic (alpha-2-adrenergic receptor agonist) | Hypertension | Extensive metabolism in the liver and intestines to several metabolites, most importantly to possible active methyldopa mono-O-sulfate (64%), methyldopamine (24%) and several conjugated and unconjugated excretory products [36] | 500-3000 mg daily | 1-5 mg/l | Orthostatic hypotension, sleepiness, sleeping problems |
| Labetalol | nonselective beta-adrenoceptor antagonist and antagonist of postsynaptic alpha 1-adrenoceptors | Hypertension | Hepatic metabolism mainly through conjugation by uridine 5'-diphospho-glucuronosyltransferase to glucuronide metabolites, without active metabolites [31] | 200-2400 mg daily | 0.015-0.1 mg/l | Hypersensitivity, orthostatic hypotension, heart failure |
| Nifedipine | dihydropyridine calcium channel blocker | Hypertension,  tocolysis | Hepatic metabolism by cytochrome P450 3A4/5 (CYP3A4/5). The limited PK data suggest that metabolism of nifedipine is more rapidly during pregnancy probably due to induction of CYP3A4/5 [38, 39] | 20-90 mg daily (hypertension)  90-110 mg daily (tocolysis) | 0.025-0.2 mg/l | Headache, peripheral edema, obstipation |
